# Impact of keel saw blade design and thickness on the incidence of tibial plateau fracture and tibial implant-loosening in cementless medial UKR

**DOI:** 10.1186/s12891-022-05500-9

**Published:** 2022-06-21

**Authors:** Lena Keppler, Steffen Klingbeil, Alexander Martin Keppler, Johannes Becker, Christian Fulghum, Björn Michel, Kilian Voigts, Wolfgang Reng

**Affiliations:** 1 BG Trauma Center Murnau, Trauma Surgery, Prof. Kuentscher Straße 8, 82418 Murnau, Germany; 2 Klinikum Garmisch-Partenkirchen, Endogap, Joint Replacement Institute, Auenstraße 6, 82467 Garmisch-Partenkirchen, Germany; 3 Department of Orthopaedics and Trauma Surgery, Musculoskeletal University Center Munich (MUM), University Hospital, LMU Munich, 81377 Munich, Germany

**Keywords:** Fracture incidence, Revision surgery, Outcome, Influence, Saw blade

## Abstract

**Background:**

Tibial plateau fractures and tibial implant- loosening are severe complications in cementless unicompartmental knee replacement (UKR). The tibial keel preparation is particularly demanding and different saw blades can be used. It was hypothesized that different blade designs and thickness have an influence on the frequency of tibial plateau fractures and implant-loosening in cementless medial UKR.

**Methods:**

1258 patients with cementless medial UKR were included in this retrospective study between 2013 and 2020. The tibial keel cut was performed either with a double keel saw blade (DKS; 2.8 mm) and added hand guided pick or a mono reciprocating saw blade (RKB) of different thickness (2.5 mm; 2.65 mm; 2.75 mm). Tibial plateau fracture and loosening were demonstrated by standard two-plane radiographs. Tibial implant-loosening was defined as complete radiolucency and implant migration. Fracture and loosening were combined with pain and loss of function.

**Results:**

In 126 patients (10%) the tibial keel was prepared with DKS, in 407 patients (32.4%) with RKB 2.5 mm, in 330 patients (26.2%) with RKB 2.65 mm and in 395 patients (31.4%) with 2.75 mm. In 4 patients (3.17%) with DKS tibial plateau fracture occurred, in 4 patients (0.99%) with 2.5 mm RKB, in 3 patients (0.92%) with 2.65 mm RKB and in 1 patient (0.25%) with 2.75 mm RKB. Significantly fewer fractures occurred with a RKB design (*p* = 0.007). A negative correlation between fracture incidence and RKB saw blade thickness was found (Spearman-*r* = − 0.93). No difference for tibial implant-loosening was shown (*p* = 0.51).

**Conclusion:**

Different blade designs and thickness have a significant influence on the incidence of tibial plateau fractures and aseptic tibial implant-loosening. The incidence of tibial plateau fractures in cementless medial UKR can be reduced by changing the design and thickness of the tibial keel saw blade. Greater thickness of RKB leads to significantly fewer tibial plateau fractures while the incidence of implant-loosening is not increasing.

Trial registration: This study was retrospectively registered and ethical approval was waived by the local ethical committee (No. 2020–1174).

## Background

Osteoarthrosis (OA) of the knee joint is one of the most common human diseases. Treatment options include total knee arthroplasty (TKA) as well as less invasive options such as unicompartmental knee joint replacement (UKR) when only one compartment is affected. Medial UKR is one of the frequently performed operations on the knee joint and is considered a safe and successful alternative to TKA [1–3]. It provides faster recovery, lower morbidity and mortality, better function and functionality [4–6]. Due to these excellent results the current literature describes an increasing need for UKR in the future [7, 8].

However, common complications occur resulting in revision surgery of UKR.

**Fig. 1:**
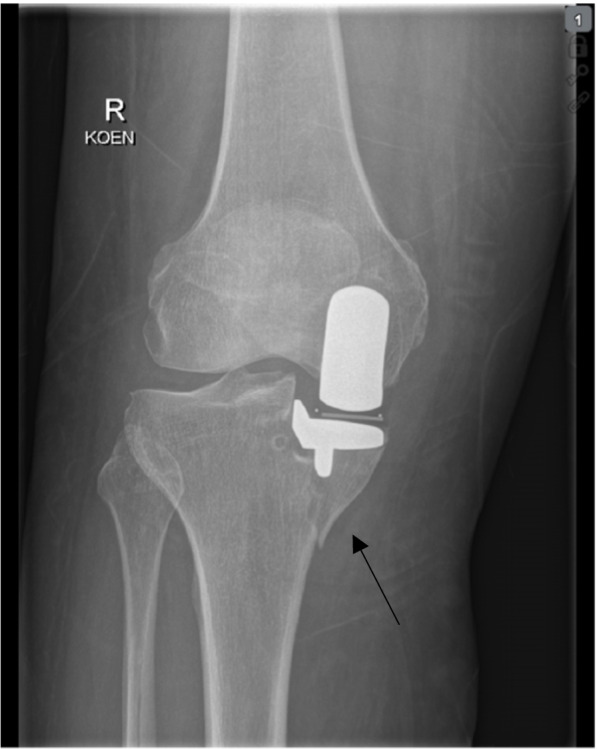
Tibial plateau fracture in a female patient after implantation of a cementless medial UKR, right knee. The fracture line can be found along the tibial keel slot (arrow)

Especially in cementless medial UKR severe complications including tibial plateau fracture and aseptic implant-loosening may occur and may require revision surgery [9–13]. In general, the incidence for tibial plateau fracture in UKR seems to be low. Depending on the evaluated studies they occur in 0.4–3.8% of all cases [14]. Up to now, there is still only limited data available [15–18]. Periprosthetic tibial plateau fractures have been associated with many risk factors such as extended sagittal tibial cut, excess removal of bone in patients with osteopenic bone, inadequate preparation of the keel slot, use of excessive force with a heavy hammer or a postoperative change in limb alignment as well as tibial shape and implant size [16–19] (Fig. 1). Patient-specific risk factors also play a significant role in both complications [20]. Cementless medial UKR provides a higher risk for tibial plateau fractures due to the impaction and the higher press fit of the different components compared to cemented UKR [12]. In both procedures the preparation of the tibia is essential. While removing the medial tibial plateau is the same, the following preparation of the tibial keel seems to be essential for the increased onset of tibial fractures in cementless UKR. In this step, a specifically designed tibial gouge is used to avoid an unnecessarily deep cut for the latter [21]. Some authors suggest that the tibial plateau fracture represents a “stress fracture” which is triggered by the vertical cut for the tibial component and results in less load-bearing and may be reduced with a parallel slot geometry [22–25]. A study by Burger et al. shows, that surgeons should be aware of excessive interference fit in cementless UKR. In combination with an impaction technique this may introduce an additional risk for tibial plateau fractures in cementless UKR. This study not only raises awareness about periprosthetic tibial fractures in cementless UKR but also highlights the importance and need for improvements in instrumentation and implants to prevent periprosthetic tibial fractures in future practices [14].

In contrast to fracture rate stands the risk for implant -loosening in medial UKR. The incidence of tibial radiolucent lines is reduced in cementless UKR [26]. While radiolucency is suggestive of reduced implant-bone contact, it is suggested that the fixation of cementless devices is at least as good, if not better than that of cemented UKR [27, 28]. Especially early after surgery tibial components in cementless UKR migrate more often than in cemented UKR due to incomplete seating or bedding-in of the component before fixation occurs [27]. Risk factors for aseptic loosening are younger age, excessive weight, and varus deformity as well as:component malalignment, undercorrection of the degeneration deformity, anterior cruciate ligament deficiency, excessive tibial slope, and bearing dislocation in mobile designs. These factors may also produce wear-induced periprosthetic osteolysis with a further increase of the component subsidence and/or loosening [29, 30].

Since many different risk factors have been postulated, the question arises as to the connection between these two severe complications in cementless medial UKR.

Although they are rare, they represent a serious issue for both the patient and the surgeon and need to be addressed. The aim of this study was therefore to investigate whether the incidence of these tibial plateau fractures depends on the design of the keel saw blade and its thickness. The hypothesis was that a thicker, optimally sized, saw blade reduces the press fit and thus the fracture incidence but does not increase the incidence of implant-loosening.

## Material and methods

In this retrospective single center study a total of 1258 cases were included in the period from January 2013–May 2020. All patients of this study suffered from isolated medial OA where the sole implantation of a medial UKR was applicable. Indication for medial UKR was strictly made according to the Oxford selection criteria. The following selection criteria played an essential role: Full cartilage loss medial, intact lateral compartment, ligamentous stability, no relevant deviation. If the patients did not meet these selection criteria cemented UKR or total knee arthroplasty (TKA) was performed. In the same period, more than 700 cemented UKR had been performed. The study was conducted in a level 1 center for joint replacement and all patients were operated by, or in supervision of, specially trained and experienced surgeons. In the study period all patients who received cementless medial unicompartmental knee replacement were included. For this knee replacement the Oxford system (Oxford partial knee, ZimmerBiomet, Warsaw, USA) was used with Oxford microplasty instruments (ZimmerBiomet, Warsaw, USA) and surgery was performed according to the manufacturer’s recommendations, besides the use of the recommended keel saw blade. Mean follow-up was three years (Maximum 7 years). All patients were evaluated clinically and radiologically regarding tibial plateau fracture and aseptic implant-loosening. Tibial plateau fracture was demonstrated by standard two-plane radiographs. Implant-loosening was defined as complete radiolucency and implant migration in two plane x-rays accompanied with pain and loss of function [31]. Each suspected case of fracture or implant-loosening was reviewed by two experienced senior surgeons of the department. Indication for revision surgery was also made by them.

To evaluate the hypothesis of the study information regarding design and thickness of the used keel saw blade for the tibial component was collected and analysed. Furthermore, epidemiological data and medical history of the affected patients were evaluated.

**Fig. 2:**
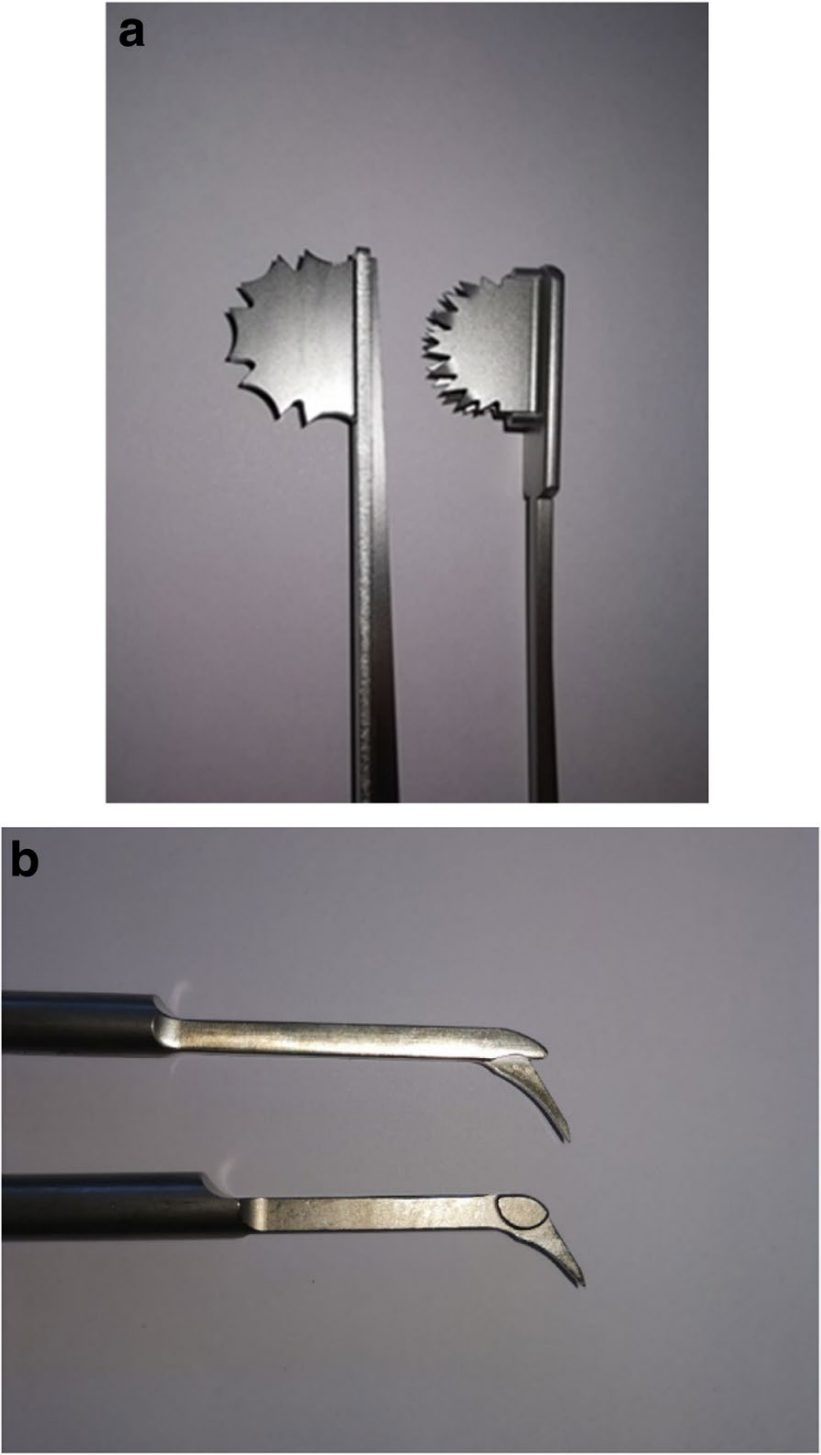
**a** Tibial keel saw blades for implantation of cementless UKR. On the left a RKB is shown. DKS on the right. DKS = Double keel saw blade, RKB = Mono reciprocating keel saw blade. **b** Image shows the bone picks which are necessary when using a DKS to remove remaining bone between the two keel slots. DKS = Double keel saw blade

According to the surgical technique manuscript for medial Oxford, the manufacturer recommends the use of a double keel saw blade (DKS) with a diameter of 2.8 mm (Fig. 2a). This saw blade has two parallel blades (Standard blade, Synvasive, ZimmerBiomet, Warsaw, USA). The preparation of the keel is started with the DKS blade and completed with a hand guided pick to remove remaining bone in the keel base. This pick has smooth sides and a sharp end (Fig. 2b). In contrast to the above described technique another design of keel saw blade, a mono reciprocating saw blade (RKB) was used in this study (Fig. 2a, Reciprocating saw blade, GominaAG, Niederwald, Switzerland). This design allows the keel preparation without the need of the hand guided pick. In this study different RKB blade thicknesses were used (2.5 mm, 2.65 mm, and 2.75 mm).

For statistical analysis Chi^2^-Test was used. Correlation was tested with Spearman-Rho (r). A level of *p* < 0.05 was determined as being statistically significant. For statistical analysis SPSS (Version 24, IBM, USA) was used.

## Results

The DKS saw blade was used in 126 patients (10%). In 407 patients (32.4%) the RKB with 2.5 mm thickness, in 330 patients (26.2%) with 2.65 mm and in 395 patients (31.4%) with 2.75 mm was used. Tibial plateau fracture occurred in 12 patients of the study group (0.95%). Mean time to fracture was 71 days after surgery.

**Fig. 3:**
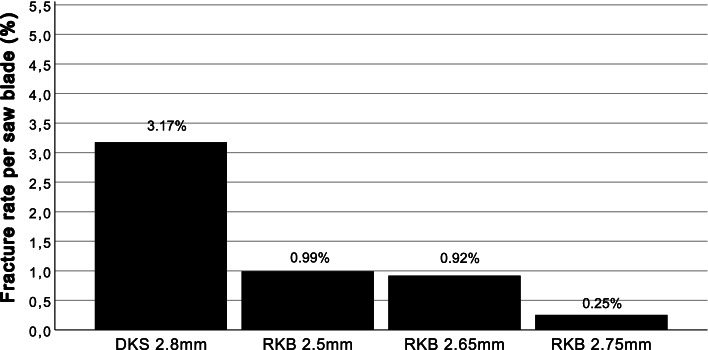
Fracture rate per saw blade. The fracture rate decreases with the use of RKB instead of DKS and decreases further with the use of a thicker RKB saw blade. DKS = Double keel saw blade, RKB = Mono reciprocating keel saw blade

Tibial plateau fracture occurred in four patients (3.17%) with the DKS saw blade. Eight tibial plateau fractures occurred in the RKB saw blade group of which four patients (0.99%) were affected after the use of a 2.5 mm RKB saw blade, three patients (0.92%) after 2.65 mm RKB and one patient (0.25%) after the use of 2.75 mm RKB (Fig. 3).

None of the patients with tibial plateau fracture had a previous trauma causing fracture. Of the affected patients, three were male and nine were female. The average age of affected patients was 73 years. Femoral component size was XS in *n* = 3 patients, S in *n* = 5, M in n = 3 and L in *n* = 1. Tibial component size was AA in *n* = 6, A in *n* = 2, B in n = 1 and size C in *n* = 3. In all patients at least one previous illness was known. Two female patients had known osteoporosis. The remaining ten affected patients had no known osteoporosis at the time of fracture. Seven of the patients suffered from heart diseases such as arterial hypertension or moderate heart failure. The remaining patients suffered from other diseases such as hypothyroidism or mild peripheral artery disease. All patients with tibial plateau fracture took at least one medication daily. None of the patients were taking corticosteroids. Mean Body Mass Index (BMI) was 29.7 kg/m^2^ (Table 1).

**Table 1:** Overview of patients’ characteristics and demographics. Most affected patients were female. BMI = Body Mass Index

	Gender (female/male)	Mean age (years)	Mean BMI (kg/m^**2**^)	Mean time to complication (days)
**Tibial plateau fracture (*n*** **= 12)**	9/3	73	29.7	71
**Implant-loosening (*n*** **= 7)**	5/2	71	30.7	309
**All patients in study (*n*** **= 1258)**	546/712	67	29.2	

All patients with tibial plateau fracture required revision surgery. In two patients conversion to TKA was necessary. In ten patients open reduction and internal fixation (ORIF) followed.

**Fig. 4:**
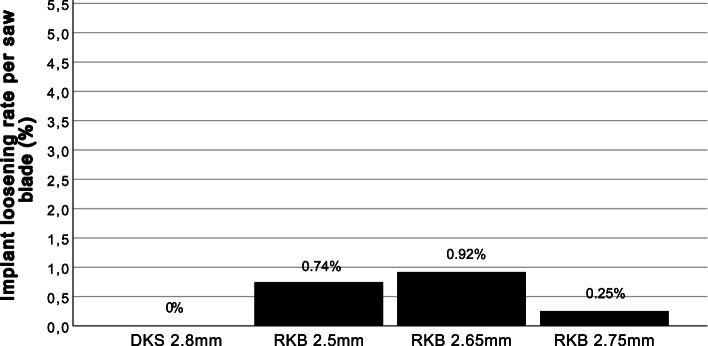
Rate of implant-loosening after use of the different keel saw blades. The rate of implant- loosening does not increase with higher thickness of saw blade. DKS = Double keel saw blade, RKB = Mono reciprocating keel saw blade

In contrast to tibial plateau fracture, implant-loosening occurred in total in seven out of the 1258 patients of this study group (0.56%). Mean time to implant-loosening after surgery was 309 days (Table 1). This complication occurred in three patients after using RKB saw blade with 2.5 mm (0.74%), in three patients with 2.65 mm (0.92%) and in one patient with 2.75 mm (0.25%). No implant-loosening occurred after DKS (Fig. 4). In no patient septic implant-loosening occurred.

Six out of the seven patients required revision surgery. In four patients conversion to TKA followed. In two patients revision surgery and implantation of a cemented tibial component was sufficient. Of the affected patients, five were female and two were male. Mean age was 71 years. Femoral component size was S in *n* = 3 patients and M in *n* = 4. Tibial component size was AA in *n* = 1, A in n = 1, B in *n* = 2, C in n = 2 and size D in *n* = 1. In six of these patients at least one previous illness was known. One female patient suffered from known osteoporosis. In the remaining affected patients, no osteoporosis was known at the time of implant-loosening. As in patients with tibial plateau fractures, most of the patients suffered from heart diseases such as arterial hypertension and coronary heart disease or hypothyroidism. All patients with implant-loosening took at least one medication daily. None of the patients were taking corticosteroids. Mean BMI was 30.7 kg/m^2^.

When comparing the two different saw blade designs (DKS vs. all RKB thicknesses) it could be shown that with the switch to RKB saw blade significantly less fractures occurred when compared to the DKS saw blade (*p* = 0.007). Four patients after DKS (3.17%) were affected compared to eight patients (0.71%) after the use of the different RKB saw blades (n total RKB = 1132). No statistically significant difference could be shown between the use of femoral or tibial implant size and a certain RKB or the DKS (*p* > 0.05) in the fracture group.

Comparing the fracture rate of the DKS group (3.17%) vs. the 2.75 mm RKB group (0.25%) it could be shown a 12.8-fold reduction of the fracture rate (*p* = 0.032).

**Fig. 5:**
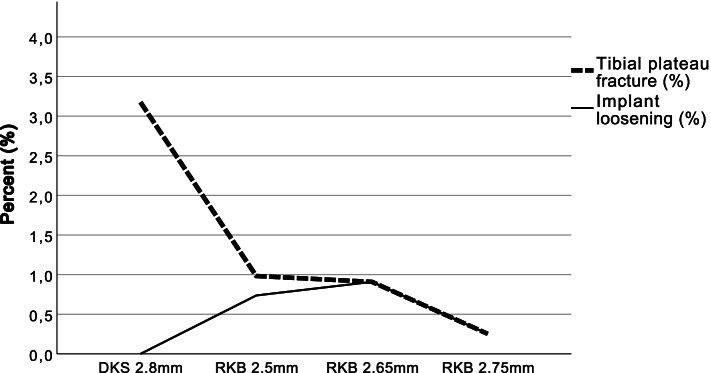
Tibial plateau fractures and implant -loosening after switching to RKB. The rate of tibial plateau fractures decreased after switching to RKB blades and decreased further with higher thickness of the used RKB. In contrast, an increasing rate of implant-loosening could not be found in the study cohort. DKS = Double keel saw blade, RKB = Mono reciprocating keel saw blade

When comparing the four saw blade groups, there was a significantly different distribution in fracture incidence (*p* = 0.035). The incidence of fractures negatively correlates significantly with higher thickness of the keel saw blade (*r* = − 0.93). The incidence of implant-loosening did not increase comparing all groups (*p* = 0.51) (Fig. 5). No statistically significant difference could be shown between the use of femoral or tibial implant size and a certain RKB or the DKS (*p* > 0.05) in the implant-loosening group.

## Discussion

To the best of our knowledge this is the first study comparing different designs and thickness of tibial keel saw blades regarding fracture incidence and occurrence of aseptic tibial implant-loosening in cementless medial UKR.

The most important finding of this study is that different saw blade designs for the tibial keel preparation in cementless medial UKR significantly influence the incidence of tibial plateau fractures. Secondly, different thicknesses by using the RKB saw blade can further reduce tibial plateau fracture incidence. Furthermore, the data reveal that the different saw blade designs have no influence on the rate of aseptic loosening of tibial components in medial UKR.

In the literature the risk of a tibial plateau fracture is higher with the cementless version than with the cemented one [12]. It is likely that the keel preparation and implant-bone interference of the keel slot are the cause for the higher incidence of fracture with the cementless version. Impaction of the keel within the walls of the slot probably causes a ‘splitting’ force in the tibia which may initiate a crack at the time of surgery [32]. Accordingly, the choice of saw blade for the tibia keel plays a major role in the occurrence of complications. The DKS saw blade appears to be disadvantageous for the individual patient and at least one RKB saw blade should be used. According to the results of the presented study, a diameter of 2.75 mm seems to be optimal to achieve the lowest possible fracture rate. This leads to the assumption that a diameter of 2.75 mm creates an optimal press fit. At the same time this size seems to be an ideal fit for the tibia keel compared to the other saw blades tested as the rate of tibial implant-loosening does not increase. Fracture rate associated with 2.75 mm RKB is more than 10 times reduced compared to DKS (Fig. 5). This result is of highest clinical relevance to avoid future tibial plateau fractures.

In our opinion, the use of the pick after tibial keel preparation with DKS assumes another relevant key factor for tibial plateau fractures. Intraoperative preparation under poor visibility is a potential risk of slipping dorsally and injuring the dorsal tibial cortex [16, 33]. Additionally, a higher press fit (interference) increases the fracture rate with DKS [19, 34]. According to Mohammed et al., an increased push-in force was necessary to insert the tibial component after DKS use (Standard blade, ZimmerBiomet, Warsaw, USA) compared to a wider and deeper blade (New blade, ZimmerBiomet), while the pull-out force remained the same [32]. Within the study, the widths for the new blade were generally more uniform throughout the depth of the slot. The slots were deeper with the new blade than the standard blade. Consequently, the use of DKS saw blades is not recommended anymore. Additionally, it could be shown in the study that fixation was not compromised with the new saw blade and therefore the rate for implant-loosening should not increase. The presented study confirms these results, as well. Furthermore, the results showed that especially older women are affected by tibial plateau fractures. Mean time to fracture was 71 days postoperatively in the presented patients and therefore correlates with the existing literature [14, 16].

To date many different factors can promote these tibial plateau fractures. One possible risk factor in this patient population (especially postmenopausal women) could be previously undiagnosed osteoporosis. In addition to osteoporosis, other patient-specific risk-factors also play an important role. Therefore, it seems essential to perform a thorough selection of patients for implantation of medial UKR as a first step. As a consequence for the clinical routine, strict selection criteria as well as consistent diagnosis and therapy should be initiated to reduce the risk of periprosthetic fractures [20, 35].

Regarding the occurrence of aseptic tibial implant-loosening, the study confirmed the existing results in the literature that older, overweight women in particular are affected by this complication [36, 37]. Additionally, it was shown that these complications occurred mainly in patients with small implant sizes. It is possible that this represents a bias, since it was mainly women who were affected. Regarding alignment in the affected patients neither preoperatively nor postoperatively were there any conspicuities, nor was there any evidence of significant abnormalities in the choice of implant size and the use of a specific saw blade and incidence of fracture and implant-loosening. It can therefore be assumed that both the alignment and implant size have no influence on our study cohort.

All patients with tibial plateau fracture required revision surgery in which either a TKA was implanted, or open reduction and internal fixation (ORIF) was performed. This shows that tibial plateau fracture is a severe complication after implantation of a medial cementless UKR.

Even with the few affected patients, it is therefore necessary to optimise the implantation procedure in order to avoid these complications as far as possible, as they are usually associated with revision surgery. As all cases with tibial plateau fracture and suspected implant-loosening were reviewed by senior surgeons, results of the interpretation and indication for revision surgery are supposed to be highly reliable.

This study has some limitations. First, the data were evaluated retrospectively. Furthermore, the rate of complications is already very low, so that statistical test procedures can only be interpreted to a limited extent. Potentially, tibial plateau fracture or substantial tibial weakening occurred intraoperatively. As none of them was registered in the routinely taken two plane radiographs intraoperatively and before discharge, we consider this possibility to be very unlikely. As surgical technique, except keel preparation, was performed in the same manner in all groups according to the manufacturer’s information and UKR has been performed for years before starting the study, we estimate the influence of a learning curve as low. To better evaluate all potential risk factors and their influence on tibial plateau fractures and tibial implant-loosening, prospective studies or even a biomechanical evaluation regarding press-fit in different saw blade thickness would certainly be useful. It would also be interesting to pay closer attention to the affected patients in order to establish a risk profile whereby all known risk factors could be taken into account.

## Conclusion

According to the results of the study, the keel saw blade design and thickness of the saw blade have an influence on the incidence of tibial plateau fracture in cementless medial UKR. This incidence decreases significantly with the use of a RKB and with higher thickness in these RKBs. Additionally, the incidence of aseptic loosening is not increasing with higher thickness of the RKB saw blade. In the future, saw blades with a thickness of e.g., 2.75 mm should therefore be used and bone preparation with DKS blades and additional hand guided pick should be avoided.

## Data Availability

All data used during this study are available from the corresponding author on reasonable request.

## Abbreviations

OA: Osteoarthritis
TKA: Total Knee Arthroplasty
UKR: Unicompartmental Knee Replacement
DKS: Double Keel Saw Blade
RKB: Reciprocating Saw Blade
BMI: Body Mass Index
